# Large-scale morphometry of the subarachnoid space of the optic nerve

**DOI:** 10.1186/s12987-023-00423-6

**Published:** 2023-03-21

**Authors:** Diego Rossinelli, Hanspeter Esriel Killer, Peter Meyer, Graham Knott, Gilles Fourestey, Vartan Kurtcuoglu, Corina Kohler, Philipp Gruber, Luca Remonda, Albert Neutzner, Jatta Berberat

**Affiliations:** 1 Institute of Neuroradiology, Kantonsspital Aarau, Tellstrasse 25, CH-5001 Aarau, Switzerland; 2 Institute of Physiology, University of Zurich, Zurich, Switzerland; 3 Department of Biomedicine, University of Basel, Basel, Switzerland; 4 Ocular Pharmacology and Physiology, University Hospital of Basel, Basel, Switzerland; 5 Biological Electron Microscopy Facility (BioEM), Swiss Federal Institute of Technology Lausanne (EPFL), Lausanne, Switzerland; 6 Scientific IT & Application Support (SCITAS), Swiss Federal Institute of Technology Lausanne (EPFL), Lausanne, Switzerland; 7 Medical Faculty, University of Bern, Bern, Switzerland; 8 Geriatric Psychiatry, Department of Psychiatry, University Hospitals of Geneva, University of Geneva, Geneva, Switzerland

**Keywords:** Cerebrospinal fluid, Subarachnoid space, Optic nerve, Normal tension glaucoma, Papilledema, Optic nerve compartment syndrome, Meningothelial cells, Arachnoid, Pia mater

## Abstract

**Background:**

The meninges, formed by dura, arachnoid and pia mater, cover the central nervous system and provide important barrier functions. Located between arachnoid and pia mater, the cerebrospinal fluid (CSF)-filled subarachnoid space (SAS) features a variety of trabeculae, septae and pillars. Like the arachnoid and the pia mater, these structures are covered with leptomeningeal or meningothelial cells (MECs) that form a barrier between CSF and the parenchyma of the optic nerve (ON). MECs contribute to the CSF proteome through extensive protein secretion. In vitro, they were shown to phagocytose potentially toxic proteins, such as α-synuclein and amyloid beta, as well as apoptotic cell bodies. They therefore may contribute to CSF homeostasis in the SAS as a functional exchange surface. Determining the total area of the SAS covered by these cells that are in direct contact with CSF is thus important for estimating their potential contribution to CSF homeostasis.

**Methods:**

Using synchrotron radiation-based micro-computed tomography (SRµCT), two 0.75 mm-thick sections of a human optic nerve were acquired at a resolution of 0.325 µm/pixel, producing images of multiple terabytes capturing the geometrical details of the CSF space. Special-purpose supercomputing techniques were employed to obtain a pixel-accurate morphometric description of the trabeculae and estimate internal volume and surface area of the ON SAS.

**Results:**

In the bulbar segment, the ON SAS microstructure is shown to amplify the MECs surface area up to 4.85-fold compared to an “empty” ON SAS, while just occupying 35% of the volume. In the intraorbital segment, the microstructure occupies 35% of the volume and amplifies the ON SAS area 3.24-fold.

**Conclusions:**

We provided for the first time an estimation of the interface area between CSF and MECs. This area is of importance for estimating a potential contribution of MECs on CSF homeostasis.

**Supplementary Information:**

The online version contains supplementary material available at 10.1186/s12987-023-00423-6.

## Introduction

**Fig. 1:**
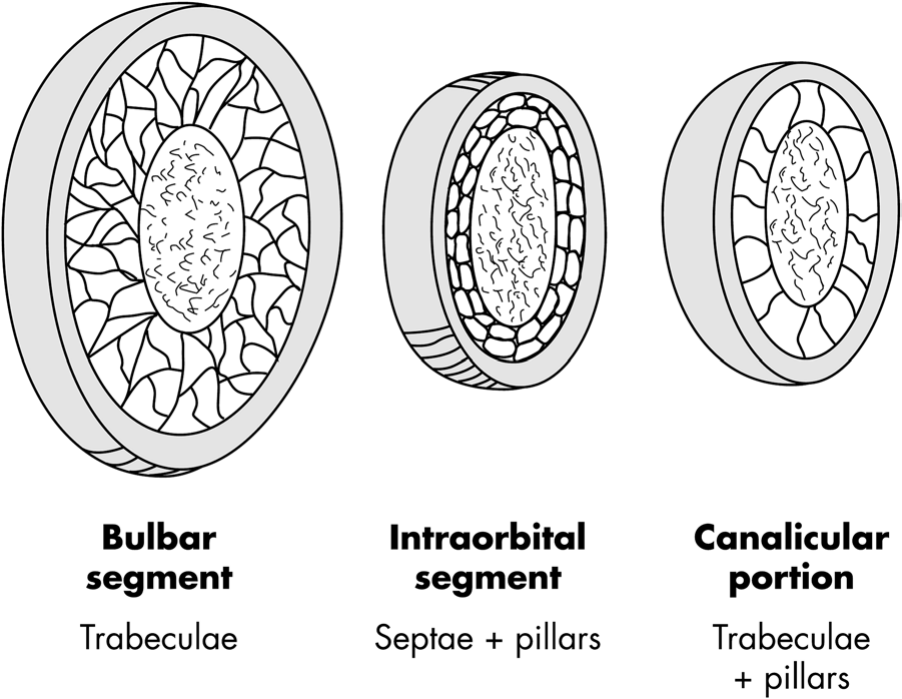
Schematic drawing of the ON SAS composition in the bulbar segment, mid-orbital segment and canalicular portion

The optic nerve (ON) is an extension of the telencephalon and as such represents a white matter tract of the central nervous system (CNS). The ON can be divided into an intracranial portion, an intracanalicular portion, and an intraorbital portion, as shown in Fig. 1 [1]. The latter is divided into a mid-orbital and a bulbar segment which is located behind the lamina cribrosa that forms a barrier between the subarachnoid space (SAS) and the eye globe. The optic nerve is wrapped into three meningeal layers: The dura mater (pachymeninges), and the leptomeninges, comprising the arachnoid mater and the pia mater. The latter is in direct contact with the optic nerve and the brain. Both the arachnoid mater and the pia mater are exposed to cerebrospinal fluid (CSF) in the SAS.

The ON SAS, just as the SAS of the brain, is bridged with a variety of trabeculae, septae, vela and pillars that extend into the arachnoid layer. Their distribution is heterogeneous along the ON: the bulbar segment contains a complex network of delicate trabeculae. The intraorbital segment contains trabeculae combined with broader septae that divide the SAS into intertrabecular fluid spaces, whereas in the canalicular portion, septae are replaced with pillars. Transmission electron microscopy (TEM) studies of the trabeculae in the bulbar segment demonstrated that the trabeculae are covered with a layer of arachnoid cells and that trabeculae are connected by thin cytoplasmic extensions, thus forming a multitude of single intertrabecular fluid spaces within the trabecular structure [1].

The dura mater harbors lymphatic capillaries that were demonstrated by TEM as well as immunohistochemistry. TEM studies show tracer particles within lymphatic clefts after injection of tracer into the SAS [2].

### Cerebrospinal fluid

Just as the ventricles, the cisterns and the cranial subarachnoid space, the SAS of the optic nerve is filled with CSF. CSF enters from the pituitary cistern via the optic canal into the ON SAS that ends blind behind the lamina cribrosa in a cul-de-sac fashion [3]. CSF offers mechanical protection to the CNS [4, 5], it supplies nutrients to the brain and the ON and acts as a clearing pathway for metabolic waste and toxic metabolites [4, 6, 7]. CSF dynamics are not yet fully understood. Next to bulk flow from production to absorption locations, there is a pulsatile component that is related to cardiac and pulmonary action [8–10]. Its main production site is the ventricular choroid plexus epithelium [5]. Historically, it has been thought that CSF is drained through the arachnoid granulations, while more recent studies suggest multiple pathways, including perineural routes and dural lymphatics [11, 12]. A possible outflow route from the optic nerve SAS are lymphatic vessels in the dura [2, 10].

### Meningothelial cells

Arachnoid and pia mater are covered by meningothelial cells (MECs), also known as leptomeningeal cells [13]. They are hypothesized to exercise a barrier function between CSF and optic nerve parenchyma, thereby contributing to CSF homeostasis: In cell cultures, they demonstrated phagocytosis of bacteria and apoptotic cell bodies, and neurotoxins, including α-synuclein and Aβ neurotoxic peptides, which are linked to Parkinson’s and Alzheimer diseases [5, 14]. MECs may also be involved in the immunological processes of the CNS, as they can secrete pro- and anti-inflammatory cytokines and chemokines [5]. MECs respond in culture to elevated CSF pressure by increased proliferation and growth [6, 15], and may thus contribute to remodeling of the SAS microanatomy in glaucoma [16].

With the MECs representing a potentially important functional exchange surface, the total area they cover is of high importance for investigating their effect on the CSF. The aim of this study is to provide a quantitative estimation of the interface area of CSF and MECs and assess a methodology to characterize compartmentalization of the ON SAS. Such quantitative analysis is of relevance to understand both CSF dynamics and pathophysiology of several disorders such a papilledema in the setting of idiopathic intracranial hypertension, axonal loss in normal tension glaucoma, among other diseases.

## Material and methods

### Sample preparation

In accordance with Swiss ethics regulations (ethics approval number EKNZ-2021–00031), a healthy human optic nerve specimen (about 6 mm x 8 mm x 10 mm) from an 85-year-old male was harvested post-mortem. Osmium tetroxide was employed for sample fixation.

### Imaging

Using synchrotron radiation-based micro computed tomography (SRµCT), we acquired datasets of the ON SAS structure at the TOMCAT beamline of the Swiss Light Source (Paul Scherrer Institute, Villigen, Switzerland). Two regions of interest of approximately 6 mm x 6 mm × 0.8 mm were imaged: Dataset1 and Dataset2. Dataset1 was obtained from a bulbar segment of the ON, and Dataset2 was acquired from an intraorbital ON segment. Overview scans with a field of view of 1.6 cm × 1.6 cm × 1.4 cm at 6.5 µm/pixels were carried out to determine the optimal imaging locations. The specimen was then imaged in-axis at 0.375 µm/pixel. Overall, we produced 71 tiles of 2560 × 2560 × 2160 pixels quantized (i.e., converted to integers) at 16bits. To facilitate image registration, a tile overlap of 20% was imposed. The total size of the acquired data was more than 2 terabytes (TBs). Image processing was carried out on the *Helvetios* supercomputer of the Swiss Federal Institute of Technology Lausanne (Lausanne, Switzerland).

### Image registration and stitching

**Fig. 2:**
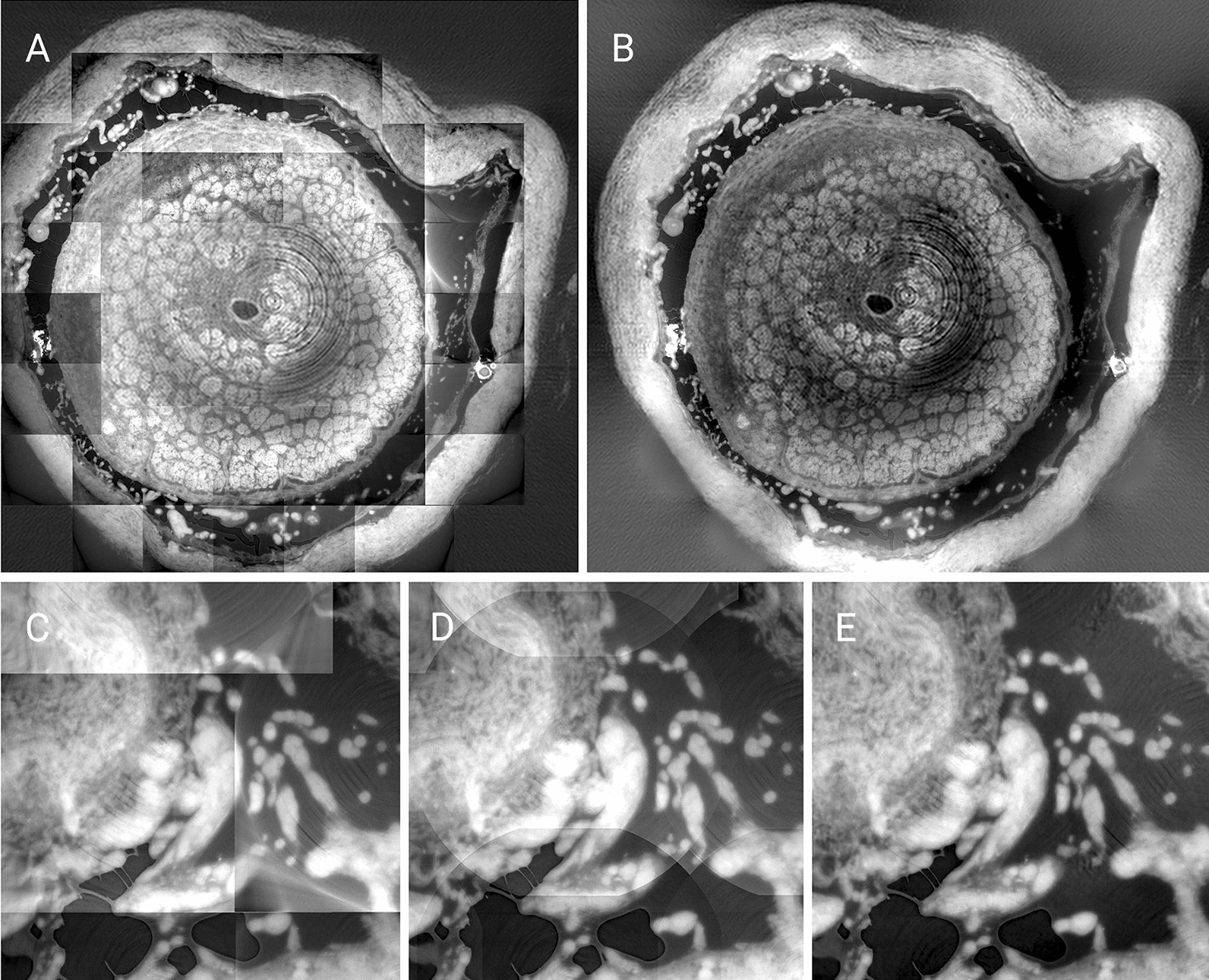
Overview of image registration (**A**) and stitching of signals at different resolutions (**B**), of Dataset2. Close-up of image registration at the field of view of a single tile (**C**), linear superposition of the signals with conventional stitching (**D**), and our stitching approach leading to an essentially artifact-free global signal (**E**)

Tile registration was carried out with a novel phase-correlation algorithm exploiting the enhanced stability of wavelet frames [17]. Due to background gradients generated by the in-axis acquisition, a direct stitching of the tile signals would lead to discontinuities. A novel in-house developed stitching algorithm based on a spectral approach was used to resample tiles in a seamless fashion and eliminate the discontinuities, as shown in Fig. 2. The stitching approach was verified against a set of prototypical grayscale signals that allow an algebraic description. Its effectiveness on real images can be assessed by consulting the Additional file 1: S8, and it has been developed specifically for the digital examinations presented in the manuscript. This approach allowed stitching together both high-resolution and coarser tiles. The reconstructed signal featured approximately 17,000 × 19,000 × 3,000 grayscale single-precision floating point pixels and an aggregate data size of more than 4* TB*.

### Image enhancement and segmentation

A hard threshold on the grayscale image would either miss the thin trabeculae (under-segmentation) or coalesce the coarsest pillars (over-segmentation). To mitigate this issue, and suppress imaging artifacts, we digitally enhanced the image with 3D continuous curvelets sampled in Fourier space [18], covering 4 octaves and 11 directions. A technical challenge is posed by the thin and elongated artifacts produced by the fixation protocol (Additional file 1: Fig. S1). The employed curvelet-based band-pass filter enables us to ignore such artifacts while contracting the intensity range of the ON SAS signal into a narrow range. A hard threshold followed image enhancement to obtain the image segments (Additional file 1: Movies S8). Morphological operations were carried out to obtain finalized CSF and ON SAS image segments.

### Connectivity analysis and manipulation

Connected components are manipulated to remove cavities in the foreground of the SAS image segment. A morphological closing allowed us to correctly capture the ON. The CSF foreground is obtained by retaining the main connected component of the inverted SAS + ON foreground. The SAS (segment) foreground, capturing only the microstructure, is obtained by a morphological closing of the CSF, intersected with the SAS + ON segment foreground (Additional file 1: Fig. S3).

### Volumetry

The volume of the space occupied by the CSF is approximated by a first order accurate discretization of $\int {U}_{\epsilon }(\phi )d\Omega$, on the entire spatial domain (i.e., over the whole image), where U_ϵ_ is a mollified unit step function and $\phi$ is the sub-pixel accurate signed Euclidean distance field of the CSF segment foreground. The area of the ON SAS trabeculae is computed by extracting a triangle mesh from the zero level-set [19] of $\phi$ at sub-pixel resolution and computing the area of the individual triangles.

### Morphometry

Morphometry calculations were carried out to measure the per-pixel model-independent 3D thickness [20, 39] of the image segments. This metric does not depend on any stereological model (e.g., rod-like, plate-like), and can be used for all varieties of shapes including trabeculae, septa and vela. Here, trabecular thickness is defined as the diameter of the maximum inscribed ball within the ON SAS microstructure. Trabecular separation is computed as trabecular thickness of the CSF segment foreground. Trabecular thickness and separation are generally thought to be computationally very expensive quantities to compute. In this work we employ a novel approach that allows to accelerate the computation 100-fold while achieving a numerical accuracy below the size of a single pixel.

## Results

**Fig. 3:**
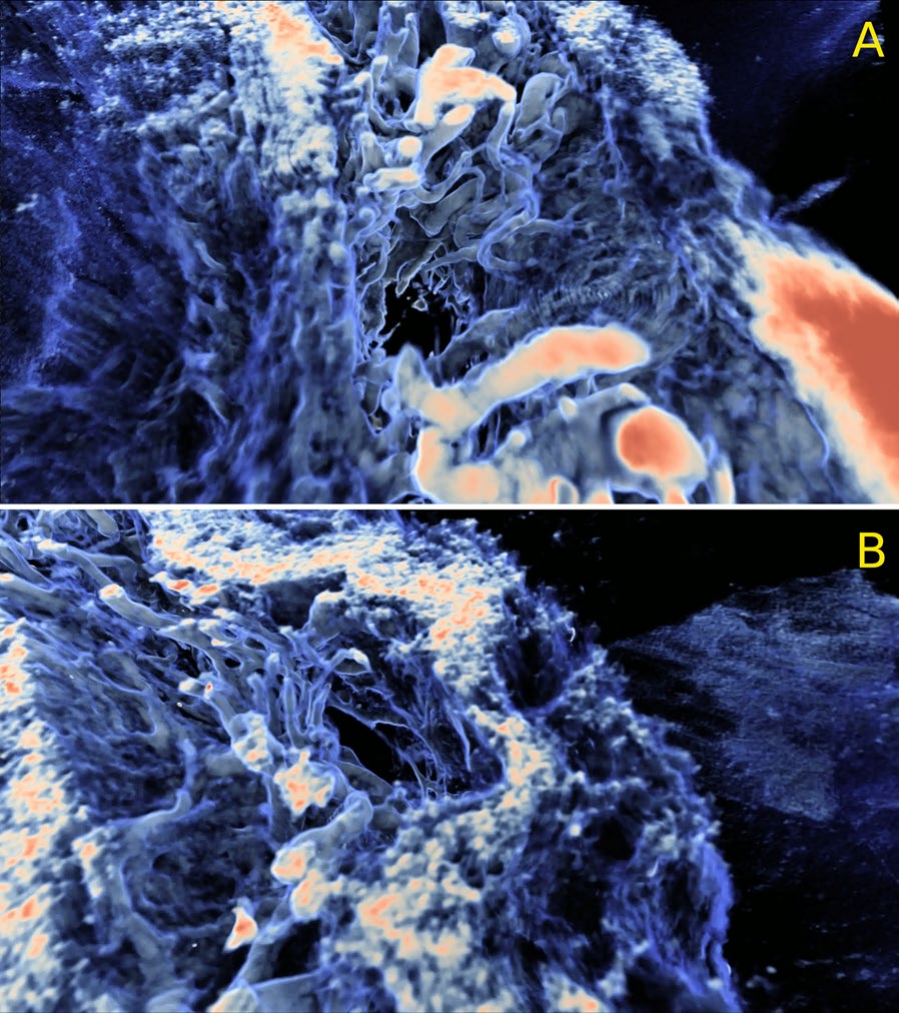
Top-view close-ups of the grayscale image showing the ON SAS, in two different regions (A and B) of the ON. Low-intensity pixels are colored in translucent blue, and high-intensity pixels are colored in opaque red

Figure 3 depicts exemplary images of the ON SAS microstructure featuring intertrabecular fluid spaces. The signal is shown to capture the details of the microstructure (see Additional file 1: S2–S9).

**Fig. 4:**
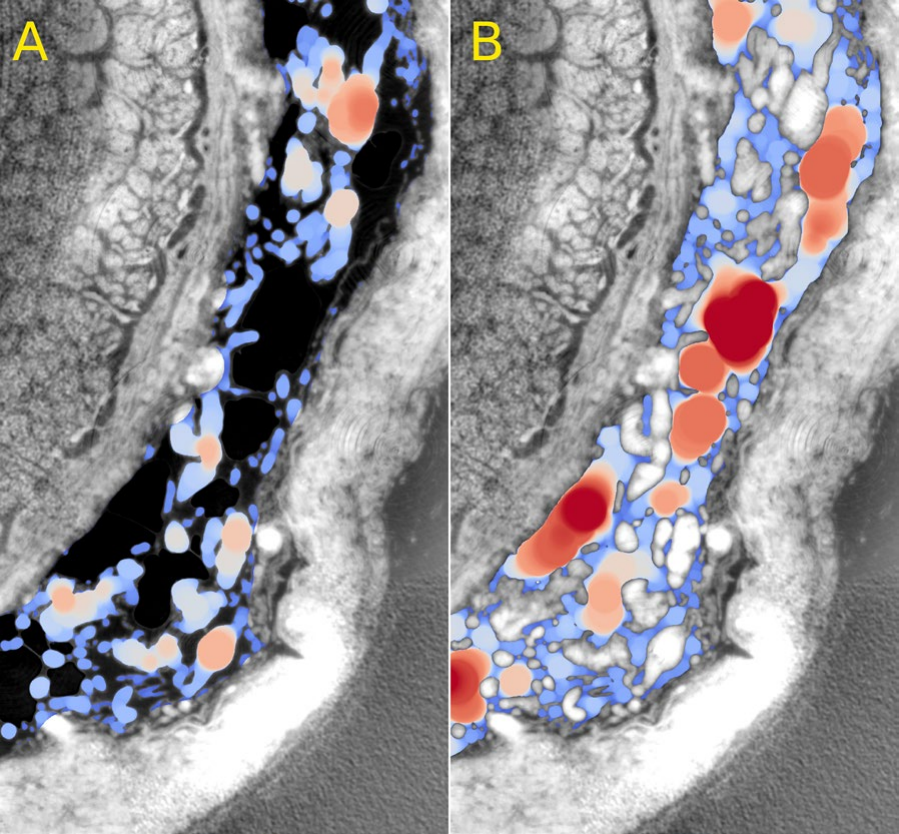
Slice close-up of 3D morphometry of *Dataset1*. Trabecular thickness (A) and trabecular separation (B) are linearly color-mapped between 2 μm (blue) to 160 μm (red)

Figure 4 depicts a slice of the global 3D morphometry of *Dataset1* featuring several intertrabecular fluid spaces with a spherical diameter of 250-500 µm. Overall, we counted 61 such spaces with a diameter larger than 130 µm and volumes between 10^−1^ and 10^−3^ mm^3^.

**Fig. 5:**
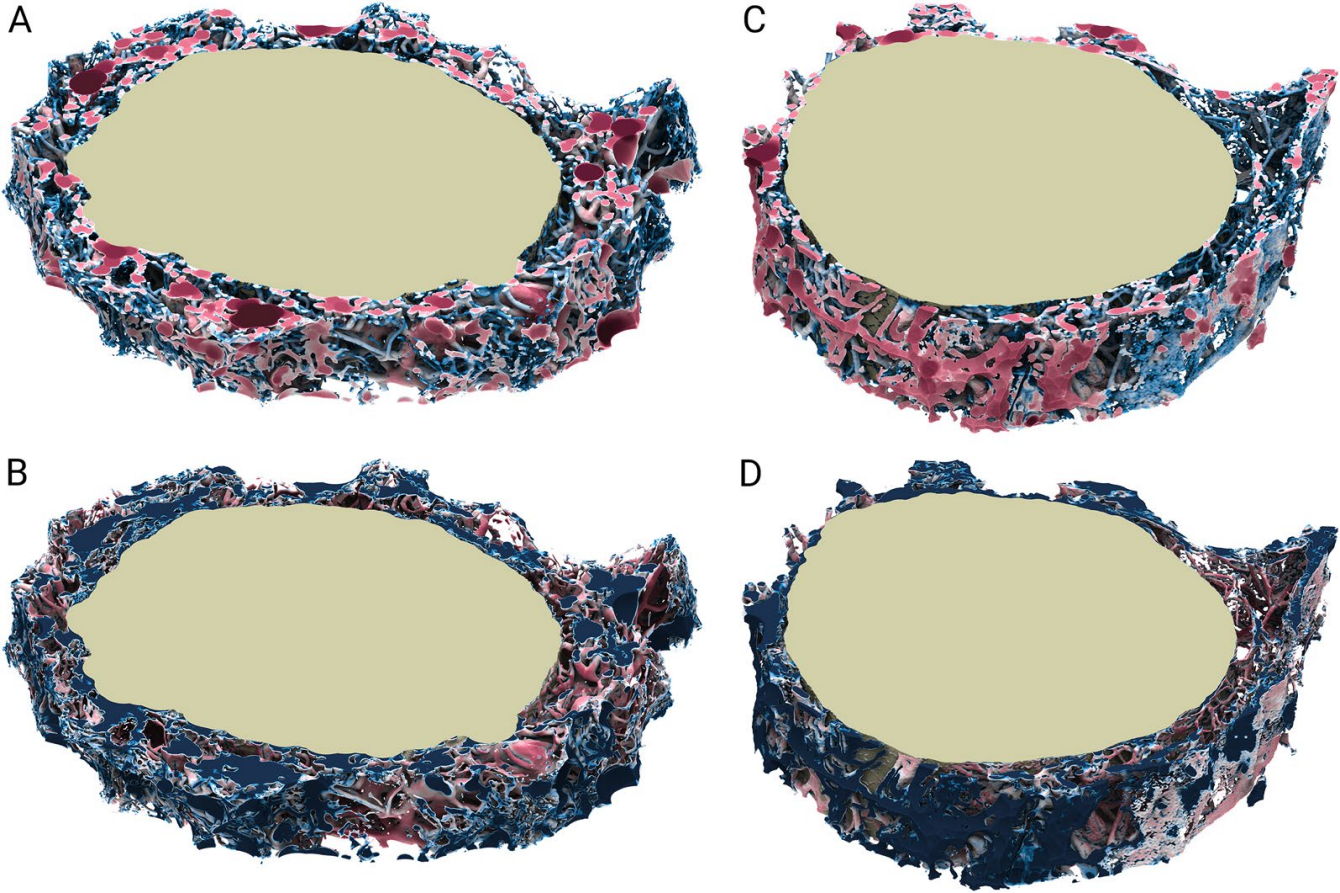
Opaque visualizations of the morphometry at the microstructure/CSF interface, with the actual ON depicted in yellow for orientation purposes. Trabecular thickness (**A**, **C**, for Dataset1, Dataset2, respectively) and trabecular separation (**B**, **D**, for Dataset1, Dataset2, respectively) between in the 0 μm–280 μm range are logarithmic color-mapped logarithmically to blue and red, respectively

Figure 5 shows a 3D opaque isosurface rendering of the ON SAS foreground/background interface (ON rendered in yellow, as reference), colored by trabecular thickness and trabecular separation. This figure conveys the heterogeneity of the trabecular thickness, as well as the intertrabecular fluid spaces.

**Fig. 6:**
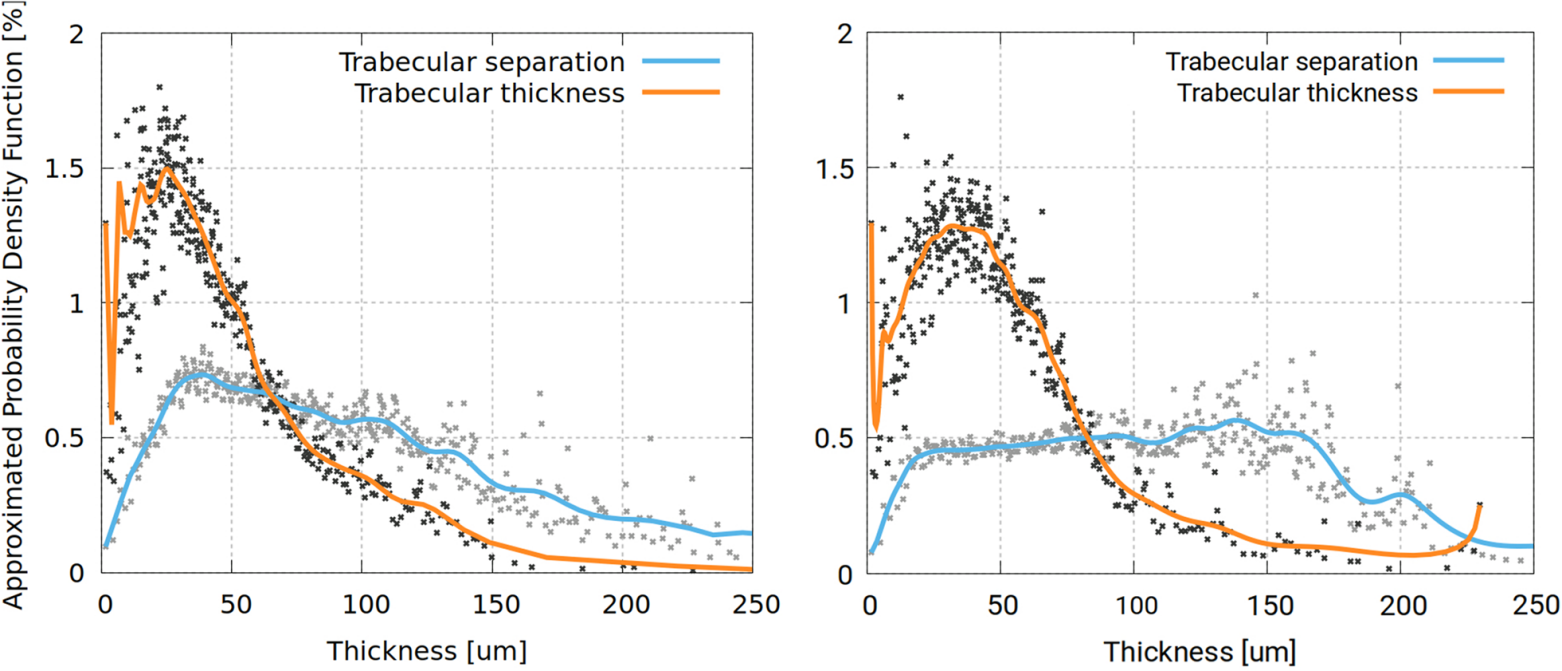
Approximated probability density functions of trabecular thickness and trabecular separation for Dataset1 (left) and Dataset2 (right)

Figure 6 shows the approximated probability density functions of trabecular thickness and trabecular separation, for both datasets. This Figure summarizes the morphometric traits of the ON SAS as it reports how the space is occupied by structures featuring heterogeneous scales. In *Dataset1, m*ost of the trabecular volume features a thickness of about 40 − 60 µm in diameter. There are no structures larger than 200 µm in diameter. Conversely, the trabecular separation density function shows that there is a substantial number of empty regions that are larger than 200 µm in diameter. These are the largest ON SAS intertrabecular fluid spaces. The distribution of trabecular thickness in the intraorbital segment is comparable to the one in the bulbar segment*.* However, trabecular separation in the bulbar and intraorbital segments are different from each other*:* in the latter, largest spaces feature a diameter in the 100 − 175 µm range, a diameter of 200 µm is reached only by 10% of intertrabecular spaces.

Table 1 reports quantities for *Dataset1,* and *Dataset2*, respectively: the length of the imaged sections, volume occupied by the CSF space, and area covered by MECs within the ON SAS. Note that area and volume entries are computed by discarding a 60-degrees circular sector where the ON was held tight during the fixation (Additional file 1: Fig. S4). To account for the missing sector, we carried out angular renormalization.

**Table 1:** Summary of the quantitative analysis of the CSF space with and without microstructure

	Dataset1		Dataset2	
	Measured	No microstructure	Measured	No microstructure
ON length	0.75 mm	0.75 mm	0.73 mm	0.73 mm
CSF space volume	2.61mm^3^	4.11mm^3^	1.68mm^3^	2.64mm^3^
ON SAS/CSF interface area	114.89mm^2^	23.65mm^2^	68.20mm^2^	21.03mm^2^

In the “No microstructure” columns, we digitally removed the ON SAS microstructure (see Additional file 1: Fig. S4 for details) to compute the entries. In *Dataset1* the microstructure occupies 35% of the ON SAS volume and amplifies the exposed surface area by a factor of 4.85. The microstructure occupies 35% of volume in *Dataset2*, too, and amplifies the MECs surface by a factor of 3.24.

## Discussion

In this study, two different sections of a healthy human optic nerve were imaged at sub-micron resolution using SRμCT. Image segmentation was preceded by large-scale image registration, stitching, and spectral enhancement. In turn, a quantitative study of morphometrics and volumetry was carried out on the image segments. The large size of the datasets necessitated image analysis on a supercomputer using custom-developed software.

Although histological studies of the human subarachnoid space of the optic nerve have been performed in the past [21], the present work reports for the first time detailed quantification of trabecular volume and trabecular surface area. Such data are of importance for the understanding of the flow dynamics of CSF within the ON SAS, as trabeculae add resistance to CSF movement [22, 23]. The data may also add important puzzle pieces for understanding the pathophysiology of disorders such a papilledema in the setting of idiopathic intracranial hypertension, axonal loss in normal tension glaucoma, as well as for the mechanism leading to the space flight associated neuro ocular syndrome [24, 25]. Compartmentalization of the subarachnoid space of the optic nerve is considered to play a role in the pathophysiology across these diseases. As trabeculae are space-occupying structures, it is conceivable that they are involved in the process of SAS compartmentalization.

The intraorbital optic nerve is divided in three different portions. The intracanalicular, the intraorbital and the retrobulbar portion. The latter is adjacent to the lamina cribrosa behind the eye globe [1]. While the subarachnoid space of the intraocular portion features broad septae, the retrobulbar portion contains a large number of delicate trabeculae similar to the ones found within the subarachnoid space of the brain [26, 27]. From a purely mechanical perspective, trabeculae and septae are building structures of the subarachnoid space that provide protection against brain trauma by acting as a viscoelastic material between the skull and the brain that can dampen the relative motion of the brain with respect to the skull [26]. Most histological studies of SAS structures, such as trabeculae, septae and velae were performed on the cranial SAS where the SAS trabeculae exercise the role of suspenders between the dura and the brain [28]. Studies of trabeculae in the SAS of the ON are scarce.

The optic nerve – a white matter tract of the central nervous system – is located for most of its length within the orbital socket that is cushioned by fat. During the wake hours, but also during REM sleep, the optic nerve is constantly in motion. Unlike the brain that is restricted in motion by the bone of the skull, the bounding tissue of the optic nerve is just the dura mater. Trabeculae and septae in the subarachnoid space of the optic nerve are structural elements that help create space for CSF and may thereby protect the optic nerve from shear damage during rapid eye movement. Given the high frequency of up to 170 000 saccades per day [29], such a protective shield is physiologically desirable. Next to their mechanical function, trabeculae and septae may play another important role: They are covered with meningothelial cells which may form a barrier towards the CSF and contribute to maintaining homeostasis of the optic nerve environment.

Our data demonstrate that the contact area between MECs lining the SAS and CSF is augmented through microstructures by factors of 3.2–4.9-fold compared to a potentially empty subarachnoid space. This is of importance as MECs play role in the homeostasis of CSF surrounding the brain and the optic nerve. Comparing surface augmentation at two sites within the bulbar region of the SAS of the optic nerve shows that the largest area of meningothelial cells is located right behind the lamina cribrosa. This is of relevance, as this area of the optic nerve is the location of the highest metabolic activity: Myelin stains demonstrate a lack of myelin around the axons at the site where they pass through the lamina cribrosa. At the same site, a high activity of ATPase is shown [30]. Axons at that site are most vulnerable to damage from toxic compounds in the CSF. Meningothelial cells at this site might be involved in the removal of toxic compounds that accumulate in a comparted SAS [14, 31, 32].

MECs were demonstrated in vitro to exercise a multitude of functions. They secrete Lipocalin-type prostaglandin D synthetase (L-PGDS) [33, 34] and IL-6 and IL-8 [35]. L-PGDS is a multifunctional protein that acts neuroprotective under normal conditions [36]. In high concentrations, as found in optic compartment syndromes, it can become toxic, and it exercises an inhibitory effect on ATP production [37]. Further, MECs were shown to release pro-inflammatory cytokines under pathophysiological conditions such as oxidative stress, increased pressure and LPS treatment [35]. In addition, MECs were shown to be capable of ingesting large amounts of bacteria, apoptotic cells bodies, amyloid-β (Aβ^1-40^) and α-synuclein thus help to maintain CSF homeostasis. The quantitative effect of MEC functions is likely to depend on their number and is therefore related to the total area they cover in the SAS. Interestingly, elevated CSF pressure has shown to decrease endocytotic activity of MECs and to cause proliferation and cell growth of meningothelial cells *in vitr*o [15]. As meningothelial cells cover all trabeculae and septae, proliferation of MECs might lead to remodeling and narrowing of the SAS and to compartmentalization and CSF sequestration with consequences for local CSF dynamics and CSF turnover. The effect of trabeculae in the ON SAS on pressure transmission along the ONSAS has been modeled mathematically [23]. More detailed quantitative information on subarachnoid space structures such as reported here may help in the development of next generation computational models.

## Limitations of the study

The subarachnoid space of the optic nerve does not only contain trabeculae but also septae that display a different geometry. This work is focused on the anatomy and geometry of the trabeculae at two locations of the retrobulbar portion of the subarachnoid space only and is, therefore, not representative for the whole SAS of the ON. Future work will include the analysis of septae, which were not present in the considered specimen.

A second limitation of the study is related to the possible morphological changes triggered by aging. The geometrical features of the ON specimen under examination may substantially differ from the ones of a younger subject. Furthermore, according to previous histological studies [40], inter-individual variability exists in the number and shape of trabeculae and septae. Generalized statements on morphology can only be achieved by considering specimens from multiple donors. The findings of this manuscript are preliminary observations that may serve as discussion starters rather than as generalizable quantifications on trabecular volume and surface area.

Thirdly, fixation of the tissues involved pinching the ON to get a stable grip and to avoid shrinkage of the tissue. This, in turn, has caused structural deformations that are visible in both datasets. We partially overcame this limitation by discarding the circular section featuring the most prominent deformations. However, as with all fixation procedures, we cannot entirely exclude the possibility of fixation artifacts.

Fourthly, while segmentation yielded high quality results for most of the dataset, over-segmentation in the ON SAS image segment upper region of the sample is apparent (Additional file 1: Fig. S2). This is explained as a side effect of the morphological closing to unite the ON with the ON SAS. Secondly, the fixation artifacts are ubiquitous in the specimen ON SAS. Many of these artifacts were, however correctly, mitigated by the image enhancement and are not part of the ON SAS foreground.

Recently, Møllgård et al. found the presence of a fourth cranial meningeal layer in the mouse brain, naming it subarachnoid lymphatic-like membrane (SLYM) [38]. We did not detect such a fourth layer in the human ON specimen investigated here. Determining the presence of SLYM required in-vivo imaging modalities, which unfortunately are precluded by the circumstances of the present work.

## Conclusion

The current study provides, for the first time, quantitative data of the volume and surface of the trabeculae in the retrolaminar portion of the subarachnoid space of the optic nerve. These data can serve as the basis for flow modeling in the ON SAS. Trabeculae do not only serve a mechanical function, but also augment the overall MEC surface area, which may be of importance for the MECs’ possible contribution to maintain homeostasis of the optic nerve.

## Supplementary Information

**Additional file 1: Figure S1**. Fixation-generated spurious artifacts in the grayscale slices, featuring strong intensity gradients and creating manifolds in the CSF (left). Issue mitigation during image enhancement and image segmentation (right) of CSF (red). **Figure S2**. High-angle views from within the grayscale images (intensities mapped from translucent blue to opaque red) showing the multi-scale features of the ON SAS microstructure. **Figure S3**. Image cuts in the z-direction (top) and x-direction (bottom) of *Dataset1*. Extended CSF (green) is obtained by engulfing the ON SAS microstructure through a morphological closing operation. **Figure S4**. Circular sectors (red) in *Dataset1 *(top) and *Dataset2 *(bottom) where CSF volume and ON SAS area have been ignored due to the structural deformation occurred during the manipulation of the specimen. **Movie S5**. Slice-by-slice movies of trabecular thickness and trabecular separation of the ON SAS foreground, both for *Dataset1 *and *Dataset2. *Blue corresponds to 2μm or less and red corresponds to 160μm. **Movie S6**. Overview movies of 3D rendering of opaque isosurfaces of trabecular thickness and trabecular separation at the ON SAS foreground/background interface, both for *Dataset1 *and *Dataset2. *Blue corresponds to 2μm or less and red corresponds to 160μm. **Movie S7**. Closeup movies showing detailed morphometry of the microstructure in the ON SAS, both for *Dataset1 *and *Dataset2. *Blue corresponds to 2μm or less and red corresponds to 160μm. **Movie S8**. Slice-by-slice movies of the stitched signals for both datasets, alongside with image segmentation of the ON SAS trabeculae (yellow) and CSF (red). **Movie S9**. Volume-rendered close-up of the stitched signal of *Dataset1*, following the CSF over the ON section.

## Data Availability

Data relative to the present document will be made available on request. Please contact the corresponding author.

## Abbreviations

ON: Optic nerve
SAS: Subarachnoid space
CSF: Cerebrospinal fluid
MEC: Meningothelial cell
